# Global Evidence of Temperature Acclimation of COVID‐19 D614G Linage

**DOI:** 10.1002/gch2.202000132

**Published:** 2021-02-15

**Authors:** Zhaonian Hao, Ruyuan Li, Chengyi Hao, Haoyuan Zhao, Xueyan Wan, Dongsheng Guo

**Affiliations:** ^1^ Department of Neurosurgery Tongji Hospital Tongji Medical College Huazhong University of Science and Technology Wuhan 430074 China; ^2^ The Second Clinical School Affiliated Tongji Hospital Tongji Medical College Huazhong University of Science and Technology Wuhan Hubei 430074 China; ^3^ Cancer Biology Research Center (Key Laboratory of the Ministry of Education) Tongji Hospital Tongji Medical College Huazhong University of Science and Technology Wuhan Hubei 430074 China; ^4^ School of Continuing Education Renmin University of China Beijing 100872 China; ^5^ State Key Laboratory of Water Resources and Hydropower Engineering Science Wuhan University Wuhan 430072 China

**Keywords:** climate impact, Covid‐19, D614G mutation, SARS‐CoV‐2

## Abstract

The novel D614G linage is becoming the dominating species of SARS‐CoV‐2. The impact of meteorological and geographical factors on SARS‐CoV‐2 pandemic are presently not well understood. This research article presents a retrospective case series. Pandemic and meteorological data from 30 countries and 49 states from USA are collected as of June 10th, 2020. The primary outcome are the coefficients of correlations between meteorological factors and pandemic data. Hierarchical clustering analysis are used on SARS‐CoV‐2 genome, meteorological factors, and pandemic. Disseminating velocity of SARS‐CoV‐2 is negatively correlated with average temperature in majority of included countries and states from USA. Proportion of the GR clade is positively associated with temperature, but is negatively correlated with altitude in countries‐set. Virus disseminating velocities in states from cluster A (Overwhelming proportion of G + GR + GH clades, GH > 60%) and C (Overwhelming proportion of G + GR + GH clades, G 20–30%) both has negative correlations with temperature, while cluster C has more significant negative correlation than cluster A. Climate and geographical environment are revealed to affect virus spreading. GH and GR clades of SARS‐CoV‐2 are probably acquiring higher temperature tolerance, while G clade may retain high temperature intolerance.

## Graphical abstract

Nowadays, the D614G linage is the dominating species of SARS‐CoV‐2. This research article suggests that climate and geographical environment could affect virus spread. GH and GR clades of SARS‐CoV‐2 are probably acquiring higher temperature tolerance, while the G clade might retain high temperature intolerance. Virus acclimation might have a critical contribution to the COVID‐19 pandemic.

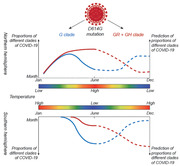

## 1 Introduction

SARS‐CoV‐2, a novel coronavirus that emerged in December 2019, has now caused a global pandemic of coronavirus disease 2019 (COVID‐19). It is still ongoing by the time of December 2020, and has caused over 70 million infection cases and 1694128 death worldwide.^[^ ^1^ ^]^ Understanding the transmission of COVID‐19 and controlling the spread out of SARS‐CoV‐2 are the most urgent affairs of international concern.

Previous researches have illustrated that SARS‐CoV‐2 could infect new cases through human‐to‐human transmission by several routes: respiratory droplets, close contact, aerosol transmission, and fecal and waterborne transmission.^[^ ^2^ ^]^ During the process of transmission of COVID‐19, environmental factors play a crucial role on influencing the viability of virus. Temperature, wind speed, humidity, ultraviolet irradiation, and air pollution are the most widely studied meteorological factors in the transmission of COVID‐19. It is reported that the half‐life of SARS‐CoV‐2 decreased from 6.3–18.6 to 1.0–8.9 h when temperature ranged from 24 to 35 °C.^[^ ^3^ ^]^ Another evidence also illustrated that the increment of ambient temperature would restrain the viabilities of respiratory coronavirus.^[^ ^4^ ^]^ However, whether higher temperatures favor or against, or uncorrelated to the transmission and severity of COVID‐19 remains controversial.^[^ ^2^, ^5^, ^6^, ^7^, ^8^, ^9^, ^10^, ^11^, ^12^, ^13^ ^]^ Humidity is also considered to have impact on the transmission of COVID‐19.^[^ ^3^, ^6^, ^8^, ^10^, ^12^, ^14^ ^]^ Recently, Carleton et al. have approved that ultraviolet radiation could decrease the growth rates of COVID‐19.^[^ ^15^ ^]^ Despite of different conclusion from early research,^[^ ^5^ ^]^ Carleton et al. provided global evidence. Wind speed and air pollution are other focuses of academic attention. Cities or areas with lower wind speed are frequently have higher level of air pollution, which has positive relation with the infection rates of COVID‐19.^[^ ^8^, ^14^, ^16^, ^17^ ^]^ These studies provide evidence to support the hypothesis that SARS‐CoV‐2 virus might bind to atmospheric particles during the process of transmission, making wind speed and air pollution both involved in the spread of COVID‐19.^[^ ^2^, ^17^, ^18^, ^19^, ^20^ ^]^ However, some researches were analyzed based on relatively smaller sample size or local area.^[^ ^6^, ^7^, ^9^ ^]^ For example, earlier studies were more focus on data from China.^[^ ^5^, ^21^, ^22^ ^]^ Moreover, some studies used the number of new cases or death cases of a day to perform analysis and didn't take incubation period of virus and accumulative effect into consideration. All these factors brought potential bias and confused the conclusion.

On the other hand, Sequencing analysis on virus genome has revealed and exhibited several significant mutations of SARS‐CoV‐2.^[^ ^23^ ^]^ One remarkable finding from latest researches that D614G mutation can enhance the infectivity by 8 to 9 folds,^[^ ^24^, ^25^ ^]^ suggesting the D614G linage and its derived clades are becoming the dominating species of SARS‐CoV‐2, which might lead to totally different epidemic than first half of 2020. Researches have suggested that D614G mutation is associated with larger virus load and increase of mortality.^[^ ^26^, ^27^ ^]^ Therefore, understanding the characteristic of D614G and its derived clades might be an essential precondition of SARS‐CoV‐2 prevention and control in the next year.

This is the first study discovering the correlations between climate, COVID‐19 pandemic, and SARS‐CoV‐2 genome clades. In the present study, we aimed to explore the underlying association between climate and COVID‐19 epidemic, and tried to verify how climate affects D614G and its derived clades of virus.

## 2 Result

### 2.1 The Relationship between COVID‐19 Epidemic and Meteorological Factors

**Figure 1:**
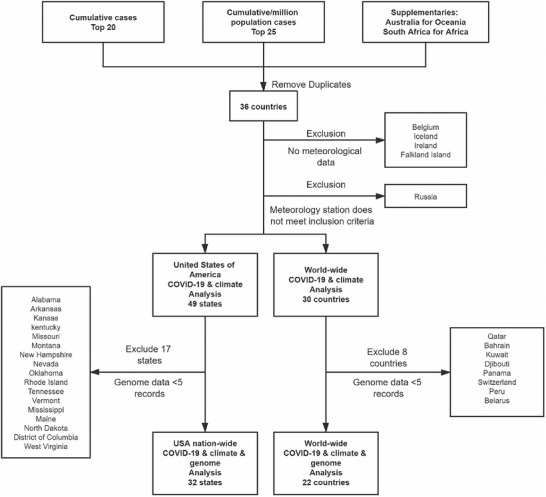
Flowchart of inclusion and exclusion. Abbreviation: COVID‐19 (Coronavirus disease 2019).

**Figure 2:**
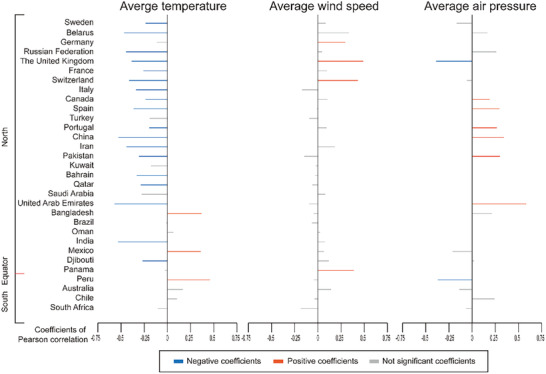
Pearson's correlation between the meteorological factors and the matched disseminating velocity of COVID‐19 worldwide (2‐week dataset). The orange and blue lines depict the positive and negative coefficients of Pearson correlation between the meteorological factors and the matched disseminating velocity of COVID‐19 with *p*‐value < 0.05. The gray lines depict the coefficients of Pearson correlation with no statistical significance (*p*‐value ≥ 0.05).

Totally, 31 countries were included to analysis the relationship of COVID‐19 pandemic and climate factors. Detailed exclusion and inclusion are shown in Methods and **Figure** **1**. Pearson analyses show significantly negative correlation of disseminating velocity of virus with average temperature of 24 h in majority of included countries. Positive correlations were also observed in some countries, mostly outside of Asia and Europe. Other tries on analyzing the associations of average wind speed and average air pressure with disseminating velocity of COVID‐19 pandemic have discovered several significant correlations in specific countries. For example, i) higher wind speed is correlated to faster spreading speed of SARS‐CoV‐2 in the United Kingdom (*r*_1w_ = 0.428, *p* < 0.001; *r*_2w_ = 0.491, *p* < 0.001, respectively) and Switzerland (*r*_1w_ = 0.201, *p* < 0.001; *r*_2w_ = 0.433, *p* < 0.001, respectively); ii) average air pressure might affect the spread of virus positively in Portugal, Pakistan (2‐week cohort), China, and the United Arab Emirates; and negatively in Peru and United Kingdom both in 1‐week and 2‐week cohort. Details of the coefficients of correlations are exhibited in **Figure** **2** and Figure S1 and Table S4 in the Supporting Information.

Furthermore, we analyzed the secondary association of coefficients of Pearson (those of temperature, wind speed, and air pressure versus virus disseminating velocity) with respect to latitudes and altitudes. Negative correlations were found between temperature coefficients and latitude (*r*_1w_ = −0.390, *p* = 0.033; *r*_2w_ = −0.385, *p* = 0.036), or altitude (*r*_1w_ = −0.315, *p* = 0.090; *r*_2w_ = −0.224, *p* = 0.234). (Table S5, Supporting Information)

**Figure 3:**
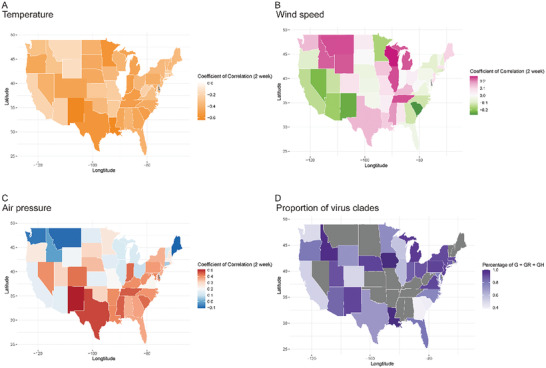
Atlas for coefficients of correlation in Pearson analysis for USA. A) Proportions of G + GR + GH clades of states in USA are shown in the map. Values of proportion are referring to color scale bar. Grey color is representing to absence of genome data in the corresponding states. B–D) The coefficients of correlation of daily meteorological estimates versus daily pandemic data are shown. B for coefficient of average air temperature in 24 h versus daily calculated disseminating velocity of virus, C and D for that of average wind speed and average air pressure respectively. Values of coefficient are referring to color scale bar.

In the analysis of USA, similar outcomes were gained in Pearson analyses that disseminating velocity of virus was generally negatively correlated with average temperature in 24 h. Air pressure was confirmed to positively affect the spread of SAR‐CoV‐2 in several states, while correlations for wind speed were only observed in a few states (details are shown in Tables S5 and S6, Supporting Information). Interestingly, in higher latitude area, the negative correlation for disseminating velocity with temperature (1‐week incubation period set) was observed to have a trend to be more obvious, but did not meet statistically significant (*r*_1w_ = −0.214, *p* = 0.140). On the other hand, the correlation coefficients for disseminating velocity with air pressure was significantly increased in higher latitude or altitude area (*r*_1w_ = −0.479, *p* < 0.001, for latitude; *r*_1w_ = −0.366, *p* = 0.010, for altitude). In order to intuitively show the potential climate and geographical impacts on COVID‐19 pandemic in USA, we generated maps with respect to clades of virus and coefficients of climate (**Figure** **3** and Figure S2: Supporting Information). Several surprising findings were marked: i) the states harboring the most significant coefficients of temperature with epidemic are mainly those along with the Cordillera mountains; ii) virus disseminating velocities in states west of the Cordillera mountains were mildly negatively correlated to wind speed, while those in Great Lakes region were generally positively correlated with wind speed, and those in states of Temperate Grassy climate region were revealed to be independent from wind speed; iii) distribution of coefficients for air pressure showed a trend of more positive in northern area and more negative in southern area of USA.

### 2.2 The Relationship between SARS‐CoV‐2 Clades and Meteorological Factors

**Figure 4:**
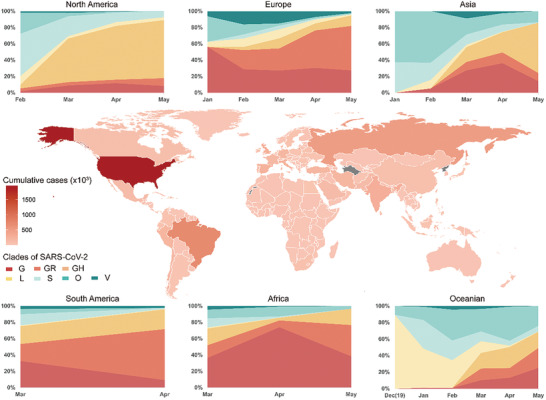
Pandemic and SARS‐CoV‐2 genome atlas globally. Global map of pandemic. The depth of filling color for each country or region is representing to cumulated cases confirmed since outbreak of COVID‐19 (As of June10th, 2020). Polygons filled with grey color are representing to absence of pandemic data from WHO reports in the corresponding countries or regions. The stacked line charts show the proportion of different clades of SARS‐CoV‐2 over time. The proportions are calculated using the sequencing data uploaded to GISAID. Detail information of virus clades are shown in Table S2 (Supporting Information). Abbreviations: SARS‐CoV‐2 (Severe acute respiratory syndrome coronavirus 2); WHO (World health Organization); GISAID (Global Initiative on Sharing All Influenza Data).

From December 2019 to February 2020, the onset of COVID‐19 pandemic, S clade and O clade were the major linages of SARS‐CoV‐2 epidemic except for that in Europe. Since March 2020, increasing viruses of G clade and G‐derived clades (GR and GH) were recorded worldwide. By June 2020, G and G derived clades have become the dominating share of pandemic except for Asia (**Figure** **4**).

Secondary Pearson analyses were performed in both countries‐set and USA‐set (Table S7, Supporting Information). Proportions of virus clades and coefficients of correlation of climate factors were enrolled to calculate the correlation index. The proportion of GR clade is positively associated with coefficient of temperature versus disseminating velocity (countries‐set, *r*_1w_ = 0.405, *p* = 0.061; *r*_2w_ = 0.406, *p* = 0.061). The proportion of G clade has significant negative correlation with coefficient (USA‐set, coefficient of temperature versus disseminating velocity, *r*_1w_ = −0.371, *p* = 0.040). Proportion of GH clade is positively associated with coefficient of temperature and wind speed (*r*_1w_ = 0.244, *p* = 0.185, for temperature; *r*_1w_ = 0.422, *p* = 0.018, for wind speed). Besides, proportion of G clade is negatively associated with coefficient of air pressure (*r*_1w_ = −0.665, *p* = 0.009).

We also analyzed the association between proportions of virus clades versus latitude or altitude (Table S8, Supporting Information). Coefficients of proportion of G clade and latitude in USA‐set is 0.390 (*p* = 0.030), respectively. Those of O clade versus altitude in countries‐set and USA‐set are 0.495 (*p* = 0.019) and 0.599 (*p* < 0.001), respectively. Indices for GR clade versus altitude in countries‐set and USA‐set are −0.442 (*p* = 0.040) and −0.200 (*p* = 0.282), respectively.

**Figure 5:**
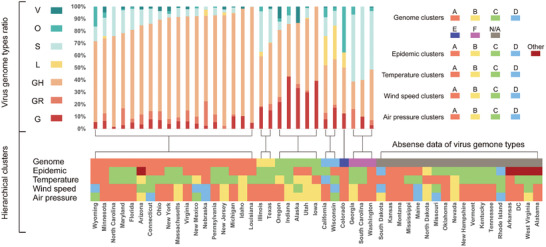
Hierarchical clustering analysis for USA. Hierarchical clustering analyses were performed individually for virus genome, daily records of pandemic, daily average temperature estimates, daily average wind speed estimates, and daily average air pressure estimates of 49 states included. The stacked bar chart shows the proportions of different clades of virus. The heat map in the below shows the clusters of factors (genome, pandemic, temperature, wind speed, and air pressure) above mentioned for corresponding state. The grey grids, labeled N/A, refer to absence of genome data. Detail information of hierarchical clusters and virus clades are shown in Table S8 (Supporting Information).

In order to make the potential association of SARS‐CoV‐2 genomes with pandemic and climate factors more explicit, we also performed hierarchical clustering analysis using genome clades data from GISAID. Through clustering analysis, states of USA were classified into 9 clusters according to epidemic disseminating velocity, 6 clusters according to virus genomes, 4 clusters for temperature variation, 5 clusters for air pressure variation, and 4 clusters for wind speed variation (**Figure** **5**). Details of clusters are shown in Table S9 (Supporting Information).

In virus genome clustering, cluster A consisted of states that have GH clade as the dominating linage (> 60%). Cluster B had over 50% proportion of total G clades (including G, GH, and GR). GH clade had the highest proportion followed by S clades. While cluster C had an overwhelming proportion of total G clades, but in which G clade had certain proportion for ≈20–30%. GH clade also accounted for the highest proportion of Cluster D and E, and followed by L or O clade, respectively. In cluster F, S clade is the majority, and GH clade is the following. Virus disseminating velocities in states from cluster A and C both had negative correlations with temperature. More particularly, cluster C had more significant negative correlation (temperature) than cluster A (median coefficient [IQR], −0.411 [−0.603 − −0.343] versus −0.292 [−0.361 − −0.206], *p* = 0.016, for 1‐week set; −0.485 vs −0.385, *p* = 0.076, for 2‐week set, Table S10, Supporting Information), which presented a more significant negative correlation for clade G than clade GH.

For temperature clustering, distribution of clusters is somehow coinciding with that of climate: a) cluster A for Temperate Deciduous Broad‐Leaved Forest climate and Subtropical Evergreen Broad‐Leaved Forest climate; b) cluster B for Temperate Desert climate and Mountain Plateau climate; c) cluster C for Temperate Savannah climate; d) cluster D for Mediterranean climate. The average temperature of cluster C is higher than cluster A (Figure S3, Supporting Information). States with genome cluster A were found to have higher proportion in the district with temperature cluster A.

## 3 Discussion

From the perspective of the relationship of between COVID‐19 epidemic and meteorological factors, our research found that the epidemic in majority of included countries were observed to negatively correlated to air temperature, indicating the virus might be limited by higher temperature to some extent, which is consist with previous studies.^[^ ^6^, ^7^, ^11^, ^14^, ^22^ ^]^ However, average wind speed and air pressure were only found to have relationship with virus dissemination in several countries and states in USA in our study. Combining all these results for wind speed, we speculated that wind speed is not a key factor of virus dissemination, which may due to the pattern of virus spreading is a close‐range fashion. This is conflict with previous researches that hypothesis the important role of wind speed in the transmission of COVID‐19.^[^ ^9^, ^12^, ^16^, ^17^, ^18^, ^20^ ^]^ There are also some reasons that might explain the disagreements between our conclusions and others that are listed below: i) analysis on climate with pandemic is based on limited sample size;^[^ ^6^, ^7^, ^9^ ^]^ ii) earlier studies that focus on data from China,^[^ ^5^, ^21^, ^22^ ^]^ in which L clade is the majority type of COVID‐19, and proportion of other clades were not adequate; iii) almost all previous studies didn't take incubation period of virus into consideration when processing data. Wu et al. did great work and found that temperature and humidity hade negative relationship with COVID‐19 new cases and new death in 166 countries with consideration of lag effect.^[^ ^12^ ^]^ However, short lag days (lag 0–3 days) might not reflect the meteorological effect properly. As Pirouz et al. found that the lag effect ranged from 2 to 8 days.^[^ ^28^ ^]^ Another potential bias the previous studies might have is directly using the number of new cases of a day to perform analysis, of which we think is not appropriate for not taking incubation period of virus and accumulative effect into consideration.

Significant correlation was found between temperature coefficients and latitude, which proposed stronger limitation that virus has in spreading as temperature increase in higher latitude or altitude area. Virus disseminating velocities in states from genome cluster A and C both had negative correlations with temperature, indicating that G and G derived clades of SARS‐CoV‐2 might able to be limited in higher temperature environment. However, when considering the different clades of SARS‐CoV‐2, the impacts of climate on epidemic were found to be distinctive.

As proportion of G clade increased, the negative correlation of temperature versus epidemic data get more significant, which might propose that G clade is more easily affected by temperature. Besides, the genome cluster C, which is contains the highest proportion of G clade, mainly matches the temperature cluster B with average temperature lower than C. According to secondary Pearson analyses, proportion of G clade is increasing as latitude goes higher in USA‐set. Thus, we propose that G clade maybe more sensitive to high temperature impact, proportion of G clade is therefore higher in lower temperature area (higher latitude area).

On the other hand, GR and GH clade might be less affected by higher temperature. In the analysis of GR clade, we found that the proportion of GR clade is positively associated with coefficient of correlation (countries‐set, temperature versus disseminating velocity). The amount of GR clade also tends to be more in low altitude environment both in countries‐set and USA‐set. As, altitude has negative correlation with temperature, these results might propose that GR clade has acquired higher temperature intolerance, so that distribution of GR clade is less affected by temperature (both high and low latitude area). The less distribution of GR clade in higher altitude area may due to other factors (for example lower air pressure). In the analysis of GH clade, GH is also positively associated with coefficient of correlation (USA‐set, temperature versus disseminating velocity). Besides, Genome cluster A, which is rich in GH clade, is mainly corresponding to temperature cluster C. As the average temperature of temperature cluster C is higher than cluster A and B, it might indicate that GH type COVID‐19 might be more adapt to higher temperature. As both GH and GR clades are derived from G clade, NS3‐Q57H and N‐G204R mutations might provide virus environment adaptation for higher temperature.

Although significant secondary correlations were identified in L, S, and V clade of virus, the absolute proportion of these clades were too low to determine the overall feature of whole pandemic. However, if the pandemic cannot be controlled rapidly, the recrudescence of G clade and L clade (outbreak in China, winter of 2019), along with potential novel mutations, is alerting in the coming autumn and winter.

## 4 Strengths and Limitations of Study

Our study selected top 20 cumulative cases countries and top 25 cumulative cases per million population countries to analyze the meteorological impact on COVID‐19 pandemic. Given that the feature of COVID‐19 pandemic was sophisticatedly influenced by numerous factors, including anti‐epidemic measures employed by government, socio‐culture factors, health system burden and so on, countries with most cumulative cases might be appropriate candidates to explore the influence of climate. We also include Australia and South Africa as supplementary countries. Considering the incubation period of virus, we matched 1‐week or 2‐weeks delayed pandemic data with the meteorological data to better describe the feature of climate impact. In addition, we use the daily new cases divided by cumulative cases to represent the disseminating velocity of COVID‐19. Since number of new confirm cases of a day is affected by baseline virus carriers, we therefore applied new diagnosed cases of that day divided by cumulated cases to lower the bias.

There are some limitations in this work. Firstly, we enrolled countries that have top confirmed cases or top confirmed cases per million population worldwide, but not all of them have both complete data of pandemic or meteorology. Second, due to huge data amount of meteorology, we can only use climate data from one city as a representation of the country to perform analysis with pandemic data. However, we chose meteorological data from the station which is nearest to the epicenter of pandemic of capital of the country to minimize the potential bias. Besides, since the genome data from GISAID are uploaded independently from whole world, not all included countries have adequate genome data to perform analysis. Therefore, the calculated proportions of different clades can only be used as approximate one in those countries without adequate sequencing data of virus. Lastly, all data, both pandemic and meteorology, in this study are recorded from winter of 2019 to summer of 2020, longer follow‐up time studies might help to support our hypothesis.

## 5 Conclusions and Policy Implications

In conclusions, GH and GR clades might have acquired the ability of higher temperature tolerance, while G clade might remain high temperature intolerance and therefore abundant in high altitude/latitude area. Climate type and geographical environment are revealed to correlated to distribution of different clades of SARS‐CoV‐2, which also features different pattern of virus spreading. Our work indicates that the evolving COVID‐19 might at least partially due to the virus acclimation, which means different clades of virus are adaptive to different environment. Applying region‐specific and season‐specific methods of virus prevention might be an essential strategy to control the epidemic. The recrudescence of G clade and potential novel clades that adaptive to lower temperature is alarming in the coming autumn and winter.

## 6 Experimental Section

##### Sample and Data

In order to unearth potential impacts of climate and geographical factors on spreading pattern of SARS‐CoV‐2 epidemic, top 20 countries worldwide were enrolled of cumulative cases and top 25 cumulative cases per million population to perform the present study (given that many of countries, which have top cumulative cases per million population, have few climate and genomic data, the sample size were enlarged from top 20 to top 25 cumulative cases per million population). Australia and South Africa were also included as supplementary countries on behalf of Oceania and Africa, respectively. Information of the included countries are shown in Table S1 (Supporting Information). After removing duplications, there are 36 countries remained. From those, Belgium, Iceland, Ireland, and Falkland Island were excluded for lacking of meteorological data. Russia was excluded due to no suitable meteorological stations available. Therefore, 31 countries were included to analysis the relationship of COVID‐19 pandemic and climate factors. In the analysis of the genomic information of COVID‐19, 13 countries were excluded for less than 50 records of COVID‐19 genome reported to Global Initiative on Sharing All Influenza Data (GISAID).

Specifically, the United States of America (USA) was chosen as an independent sample for the reasons listed below: 1). USA has wide ranges of longitude and latitude with diverse climate type, enabling the analysis for the relationship of COVID‐19 pandemic and meteorological factors inter and intra states of USA; 2). The number of cumulative cases of COVID‐19 in USA has been preceding those of other countries. Meanwhile, public health measures, such as social distance and government advice for mask‐wearing, are relatively insufficient, making the virus transmission in USA a more natural status with little human intervention. And this enables to perform a more reliable analysis; 3). Most states in USA have abundances of genomic data of COVID‐19 with integrated climate data, making the analysis practicable. Therefore, USA was chosen as a kind of sensitive analysis. In total, data from 49 states were included to analyze relationship of COVID‐19 pandemic and climate factors. In which 17 states were excluded for less than 5 records of COVID‐19 genome records.

Pandemic data of COVID‐19 cases were collected from World Health Organization Coronavirus COVID‐19 situation reports^[^ ^1^ ^]^ as of June 10th, 2020 for analysis. Climate and geographical data of each country were collected from China Meteorological Data Service Center (CMDC),^[^ ^29^ ^]^ For each country, data were collected from the monitoring stations nearest to each country's capital or the city with the largest number of confirmed cases. Detailed information of the chosen monitoring stations is summarized in Table S3 (Supporting Information). For each state in the USA, data were collected from the monitoring stations of the state. Genomic information of SARS‐CoV‐2 were extracted from GISAID as of June 10th, 2020. The clades of SARS‐CoV‐2 has been divided into S, L, V, G, GH, GR, and O clades according to GISAID.^[^ ^30^ ^]^ The alterations and detailed information of these clades are showed in Table S2 (Supporting Information).

##### Measures of Variables

Considering that the dissemination of the virus is associated with the population base of the confirmed cases, the disseminating velocity of pandemic of a day is defined in each country or states as daily new diagnosed cases of that day divided by cumulated cases of the day before.

(1) $$\begin{array}{c} \mathrm{Disseminating}\;\text{velocity}\;\text{of}\;a\;\text{day}=\frac{\text{Daily}\;\text{new}\;\;\text{cases}\;\text{of}\;\text{the}\;\text{day}}{\text{Cumulated}\;\text{cases}\;\text{of}\;\text{the}\;\text{day}\;\text{before}}\;\times 100\% \end{array}$$

The impact of meteorological factors was considered on the spread of virus could not be reflected by the same day of new confirmed cases, because of the time delays due to incubation period of virus. Besides, the period from symptoms onset to laboratory confirmation also contributes to the delay. Since the incubation period of COVID‐19 was reported 3–10 days with a median incubation period of 7 days and the medical observation period are 14 days,^[^ ^31^ ^]^ 1‐week or 2‐weeks delayed pandemic data was used to match the meteorological data. 1‐week dataset and 2‐week dataset are analyzed as sensitive analysis.

Disseminating velocity of Day A matches Meteorological data of Day (A−7)
Or Disseminating velocity of Day A matches Meteorological data of Day (A−14)

##### Data Analysis

Pearson correlation was applied to perform correlation analysis in this work. Hierarchical clustering is used for clustering analysis, and coefficient of correlation is applied for metric of clustering calculation. Medians of continuous variables that were not normally distributed were compared using the Wilcoxon rank‐sum test. All statistical tests were 2‐tailed, and statistical significance was defined as α less than 0.1. All statistical analyses are performed in SPSS version 22. Figures are plotted using R.

## Conflict of Interest

The authors declare no conflict of interest.

## Author Contributions

Z.H. and R.L. contributed equally to this work. Z.N.H. conceptualized and designed the study. Z.N.H. and R.Y.L. drafted the manuscript. Z.N.H., R.Y.L., and H.Y.Z. collected pandemic and meteorological data and double‐checked. C.Y.H. finished statistical analyses. Z.N.H. and R.Y.L. analyzed and interpreted the results. D.S.G. and X.Y.W. advised on conception and design of the study. D.S.G. revised the manuscript and offered funding and guidance. All authors vouch for the respective data and analysis, revised, approved the final version and agreed to publish the manuscript. The corresponding author attests that all listed authors meet authorship criteria and that no others meeting the criteria have been omitted.

## Supporting information

Supporting Information

## Data Availability

Data used in this research were available in the public domain: https://www.gisaid.org/. No additional data available.
