# Supplementary material for: Global Evidence of Temperature Acclimation of COVID‐19 D614G Linage
Source: Glob Chall. 2021 Feb 15;5(6):2000132. doi: 10.1002/gch2.202000132 (PMC7995217; doi:10.1002/gch2.202000132)
Supplement: Supplementary file 1 — Supporting Information [file GCH2-5-2000132-s001.pdf]

© 2021 Wiley-VCH GmbH

Global Challenges
Open Access

Supporting Information

for *Global Challenges*, DOI: 10.1002/gch2.202000132

Global Evidence of Temperature Acclimation of COVID-19 D614G Linage

*Zhaonian Hao, Ruyuan Li, Chengyi Hao, Haoyuan Zhao, Xueyan Wan, and Dongsheng Guo**

© Copyright 2021. Wiley-VCH GmbH

Supplementary Appendix

Supplementary Tables

Supplementary Table 1. Information of Included Countries (as of June 10th, 2020)

Supplementary Table 2. Information of COVID-19 Clades Classification

Supplementary Table 3. Information of Selected Stations of Each Country and Each State of USA

Supplementary Table 4. Pearson's correlation between the meteorological factors and the matched disseminating velocity of COVID-19 worldwide

Supplementary Table 5. Secondary Pearson's Correlation between Coefficients of Climate Factors and Altitude/Latitudes

Supplementary Table 6. Pearson's correlation between the meteorological factors and the matched disseminating velocity of COVID-19 in USA

Supplementary Table 7. Secondary Pearson's Correlation between COVID-19 Clades and Coefficients of Climate Factors

Supplementary Table 8. Secondary Pearson's Correlation between COVID-19 Clades and Altitude/Latitudes

Supplementary Table 9. Cluster Analysis of COVID-19 Clades, Pandemic, Average Temperature, and Average Wind Speed in USA

Supplementary Table 10. Intra-cluster Analysis in Epidemic Disseminating Velocity Cluster and COVID-19 Genome Cluster in USA

Supplementary Figures

Supplementary Figure 1. Pearson's correlation between the meteorological factors and the matched disseminating velocity of COVID-19 worldwide (1-week dataset)

Supplementary Figure 2. Atlas for distributions of virus clades for USA and Atlas for coefficients of correlation in Pearson analysis for USA (1-week delayed matching of data)

Supplementary Figure 3. Line chart for temperature cluster A and C

Supplementary Table 1. Information of Included Countries (as of June 10th, 2020)[1]

Countries with Top 20 Cumulative Cases of COVID-19	
Country	Cumulative Confirmed Cases
The United States of America	2000464
Brazil	772416
Russia	501800
The United Kingdom	291588
India	286576
Spain	242280
Italy	235763
Peru	208823
France	192068
Germany	186525
Iran	180156
Turkey	173036
Chile	148456
Mexico	129184
Pakistan	119536
Saudi Arabia	112288
Canada	98720
China	84210
Bangladesh	78052
Qatar	75071
**Countries with Top 25 Cumulative Cases Per Million Population of COVID-19**	
Country	Cumulative Confirmed Cases/million
Qatar	25545
Bahrain	9793
Kuwait	7919
Chile	7768
Peru	6179
USA	5947
Belarus	5320
Iceland	5299
Spain	5175
Belgium	5128
Ireland	5106
Sweden	4547
Djibouti	4426
Falkland Island	4333
The United Kingdom	4248
The United Arab Emirates	4096
Panama	3994
Italy	3896
Oman	3698
Switzerland	3571
Brazil	3479
Portugal	3462
Russia	3383
Saudi Arabia	3225
**Supplementary Countries**	
Country	Cumulative Confirmed Cases
Australia	7267
South Africa	52991

Abbreviations: COVID-19, coronavirus disease 2019.

Supplementary Table 2. Information of COVID-19 Clades Classification[2]

COVID-19 Clades	Nucleic Acid Alteration	Protein Alteration
S	C8782T, T28144C	NS8-L84S
L	C241, C3037, A23403, C8782, G11083, G25563, G26144, T28144, G28882	/
V	G11083T, G26144T	NSP6-L37F + NS3-G251V
G	C241T, C3037T, A23403G	S-D614G
GH	C241T, C3037T, A23403G, G25563T	S-D614G + NS3-Q57H
GR	C241T, C3037T, A23403G, G28882A	S-D614G + N-G204R
O (Others)	/	/

Abbreviations: COVID-19, coronavirus disease 2019.

Supplementary Table 3. Information of Selected Stations of Each Country and Each State of USA.

**Worldwide**		
**Country**	**Selected Station**	**Reason**
Australia	Sydney	Capital
Bahrain	Bahrain International Airport	The only station of Bahrain
Bangladesh	Dhaka	Capital, center of the country
Belarus	Minsk	Capital, center of the country
Brazil	Santos	Congested area, close to Saint Paul/Rio de Janeiro/Curitiba
Canada	Toronto	Capital, most populous area nationwide
Chile	Quinta Normal	In Santiago, the biggest city in Chile, center of the country
China	Wuhan	Initial outbreak and the most severely affected city in China
Djibouti	Djibouti	Capital
France	Paris	Capital
Germany	Hannover	One of the most severely affected cities in Germany, center of the country
India	Bombay	The most populous city, the most severely affected city in India
Iran	Tehran	The biggest city and the most severely affected city in Iran
Italy	Milano	The second biggest city and one of the most severely affected cities in Italy
Kuwait	Kuwait International Airport	The only station of Kuwait
Mexico	Mexico D. F	Capital
Oman	Muscat International Airport	Capital, the biggest city in Oman
Pakistan	Lahore City	The second biggest city and the most severely affected city in Pakistan
Panama	Panama City	Capital
Peru	Lima-Callao International Airport	Capital, the biggest city in Peru
Portugal	Lisbon	Capital
Qatar	Doha International Airport	Capital
Saudi Arabia	Riyadh	Capital, the biggest city in Saudi Arabia
South Africa	Cape Town International Airport	The second biggest city in South Africa
Spain	Madrid	Capital
Sweden	Stockholm	Capital
Switzerland	Zurich	Capital
The United Kingdom	London	Capital
Turkey	Istanbul	Capital, the biggest city and the most severely affected city in Turkey,
The United Arab Emirates	Abu Dhabi	Capital
**States of USA**		
**States**	**Selected Station**	
Alabama	Montgomery	
Alaska	Anchorage	
Arizona	Phoenix	
Arkansas	Fort Smith	
California	Los Angeles	
Colorado	Denver	
Connecticut	Hartford	
Florida	Jacksonville	
Georgia	Atlanta	

Idaho	Boise
Illinois	Chicago
Indiana	Indianapolis
Iowa	Des Moines
Kansas	Topeka
Kentucky	Louisville
Louisiana	New Orleans
Maine	Portland
Maryland	Baltimore
Massachusetts	Boston
Michigan	Detroit
Minnesota	Minneapolis
Mississippi	Jackson
Missouri	Kansas City
Montana	Billings
Nebraska	Omaha
Nevada	Las Vegas
New Hampshire	Concord
New Jersey	Newark
New Mexico	Albuquerque
New York	New York City
North Carolina	Charlotte
North Dakota	Fargo
Ohio	Columbus
Oklahoma	Oklahoma City
Oregon	Portland
Pennsylvania	Philadelphia
Rhode Island	Providence
South Carolina	Columbia
South Dakota	Sioux Falls
Tennessee	Memphis
Texas	Houston
Utah	Salt Lake City
Vermont	Burlington
Virginia	Richmond
Washington	Seattle
Washington District of Columbia	Washington District of Columbia
West Virginia	Charleston
Wisconsin	Milwaukee
Wyoming	Cheyenne

Supplementary Table 4. Pearson's correlation between the meteorological factors and the matched disseminating velocity of COVID-19 worldwide

	Meteorological Variables											
Country	Average Temperature (1 Week)	P value[a] (1 Week)	Average Temperature (2 Week)	P value[a] (2 Week)	Average Wind Speed (1 Week)	P value[a] (1 Week)	Average Wind Speed (2 Week)	P value[a] (2 Week)	Average Air Pressure (1 Week)	P value[a] (1 Week)	Average Air Pressure (2 Week)	P value[a] (2 Week)
Australia	0.223	0.009	0.165	0.054	0.014	0.876	0.141	0.098	-0.104	0.231	-0.138	0.106
Bahrain	-0.172	0.283	-0.330	0.035	-0.143	0.373	-0.025	0.877				
Bangladesh	0.446	0.005	0.371	0.022	0.013	0.937	-0.041	0.805	0.202	0.224	0.213	0.199
Belarus	-0.554	0.001	-0.466	0.008	0.071	0.736	0.335	0.101	0.233	0.207	0.167	0.371
Brazil	0.246	0.023	-0.011	0.918	0.251	0.020	-0.059	0.576				
Canada	-0.180	0.053	-0.236	0.009	0.111	0.237	0.103	0.255	0.141	0.132	0.190	0.035
Chile	0.188	0.166	0.104	0.445	0.288	0.031	-0.031	0.819	-0.135	0.321	0.240	0.075
China	-0.480	<0.001	-0.526	<0.001	-0.065	0.423	0.005	0.949	0.375	<0.001	0.343	<0.001
Djibouti	-0.226	0.050	-0.267	0.015	-0.021	0.860	0.120	0.918	0.032	0.784	0.022	0.843
France	-0.305	<0.001	-0.257	0.002	0.148	0.086	0.099	0.247	-0.007	0.935	0.003	0.969
Germany	-0.354	<0.001	-0.112	0.221	0.114	0.229	0.296	0.001				
India	-0.334	0.015	-0.532	<0.001	-0.069	0.625	0.077	0.584				
Iran	-0.600	<0.001	-0.443	<0.001	-0.027	0.797	0.184	0.068				
Italy	-0.319	0.001	-0.338	<0.001	-0.162	0.087	-0.167	0.069				
Kuwait	0.069	0.668	-0.177	0.269	-0.134	0.404	-0.016	0.919				
Mexico	0.335	0.026	0.362	0.016	-0.044	0.778	0.067	0.665	-0.216	0.164	-0.209	0.179
Oman	-0.431	0.008	0.064	0.707	-0.191	0.257	0.025	0.885				
Pakistan	-0.287	0.032	-0.305	0.022	-0.167	0.219	-0.142	0.296	0.224	0.103	0.302	0.027
Panama	-0.105	0.418	-0.028	0.848	0.212	0.140	0.389	0.005				
Peru	0.303	0.029	0.460	0.001	0.040	0.777	-0.042	0.767	-0.388	0.005	-0.369	0.009
Portugal	-0.260	0.010	-0.196	0.050	-0.041	0.682	0.096	0.341	0.236	0.050	0.268	0.007
Qatar	-0.443	0.001	-0.288	0.045	0.188	0.196	-0.054	0.712				
Saudi Arabia	-0.466	0.001	-0.279	0.064	-0.160	0.293	0.080	0.603				
South Africa	0.131	0.335	-0.106	0.435	0.091	0.503	-0.183	0.178	-0.787	0.063	-0.067	0.899
Spain	-0.301	0.001	-0.264	0.002	0.109	0.220	-0.008	0.929	0.169	0.056	0.295	0.001
Sweden	-0.267	0.002	-0.236	0.006	0.016	0.855	0.084	0.337	-0.137	0.121	-0.165	0.058
Switzerland	-0.443	<0.001	-0.414	<0.001	0.201	0.041	0.433	<0.001	0.055	0.576	-0.057	0.559
The United Kingdom	-0.395	<0.001	-0.386	<0.001	0.428	<0.001	0.491	<0.001	-0.301	0.002	-0.386	<0.001
Turkey	-0.207	0.084	-0.189	0.098	-0.785	0.535	-0.089	0.439				
The United Arab Emirates	-0.533	<0.001	-0.569	<0.001	-0.072	0.466	-0.088	0.366	0.559	<0.001	0.583	<0.001

Average temperature (1/2 Week) indicates the Pearson's correlation between daily average temperature (℃) within 24 hours and the matched 7-day-delayed/14-day-delayed disseminating velocity of COVID-19. Average wind speed (1/2 Week) indicates the Pearson's correlation between daily average wind speed (m/s) within 24 hours and the matched 7-day-delayed/14-day-delayed disseminating velocity of COVID-19. Average air pressure (1/2 Week) indicates the Pearson's correlation between daily average air pressure (hPa) within 24 hours and the matched 7-day-delayed/14-day-delayed disseminating velocity of COVID-19.

Abbreviations: COVID-19, coronavirus disease 2019.

[a] P values indicate differences of Pearson correlations. $P < 0.1$ was considered statistically significant.

Supplementary Table 5. Secondary Pearson's Correlation between Coefficients of Climate Factors and Altitude/Latitudes.

	Latitude		Altitude	
	Correlation	P value[a]	Correlation	P value[a]
**Worldwide**				
Average Temperature (1 Week)	-0.390	0.033	-0.315	0.090
Average Temperature (2 Week)	-0.385	0.036	-0.224	0.234
Average Wind Speed (1 Week)	0.101	0.594	0.076	0.691
Average Wind Speed (2 Week)	0.286	0.126	0.159	0.402
Average Air Pressure (1 Week)	0.053	0.833	0.135	0.592
Average Air Pressure (2 Week)	0.042	0.868	0.295	0.235
**The United States of America**				
Average Temperature (1 Week)	-0.214	0.140	0.003	0.984
Average Temperature (2 Week)	0.002	0.990	0.014	0.923
Average Wind Speed (1 Week)	0.001	0.997	-0.100	0.493
Average Wind Speed (2 Week)	0.211	0.145	0.035	0.810
Average Air Pressure (1 Week)	-0.479	<0.001	-0.366	0.010
Average Air Pressure (2 Week)	-0.662	<0.001	0.029	0.843

Average temperature (1/2 Week) indicates the Pearson's correlation between daily average temperature (℃) within 24 hours and the matched 7-day-delayed/14-day-delayed disseminating velocity of COVID-19. Average wind speed (1/2 Week) indicates the Pearson's correlation between daily average wind speed (m/s) within 24 hours and the matched 7-day-delayed/14-day-delayed disseminating velocity of COVID-19. Average air pressure (1/2 Week) indicates the Pearson's correlation between daily average air pressure (hPa) within 24 hours and the matched 7-day-delayed/14-day-delayed disseminating velocity of COVID-19.

[a] P values indicate differences of Pearson correlations. $P < 0.1$ was considered statistically significant.

Supplementary Table 6. Pearson's correlation between the meteorological factors and the matched disseminating velocity of COVID-19 in USA

	Meteorological Variables											
States	Average Temperature (1 Week)	P value[a] (1 Week)	Average Temperature (2 Week)	P value[a] (2 Week)	Average Wind Speed (1 Week)	P value[a] (1 Week)	Average Wind Speed (2 Week)	P value[a] (2 Week)	Average Air Pressure (1 Week)	P value[a] (1 Week)	Average Air Pressure (2 Week)	P value[a] (2 Week)
Alabama	-0.247	0.047	-0.382	0.002	0.650	<0.001	0.360	0.004	-0.078	0.535	0.060	0.637
Alaska	-0.71	<0.001	-0.728	<0.001	0.211	0.094	-0.083	0.515	-0.180	0.391	0.087	0.493
Arizona	-0.219	0.020	-0.216	0.022	0.206	0.029	0.151	0.111	-0.155	0.101	-0.183	0.053
Arkansas	-0.253	0.042	-0.257	0.039	0.373	0.002	0.406	0.006	0.118	0.350	0.030	0.814
California	-0.13	0.171	-0.200	0.034	0.230	0.014	0.159	0.092	-0.050	0.599	-0.144	0.127
Colorado	-0.267	0.021	-0.484	<0.001	0.115	0.329	0.432	<0.001	0.002	0.785	0.074	0.530
Connecticut	-0.082	0.502	-0.167	0.170	0.185	0.131	0.057	0.642	0.126	0.304	-0.095	0.440
DC	0.201	0.117	-0.023	0.859	0.426	0.001	0.135	0.300	0.136	0.292	0.089	0.494
Florida	-0.318	0.005	-0.405	<0.001	0.328	0.004	0.372	0.001	-0.071	0.542	-0.037	0.751
Georgia	-0.35	0.002	-0.467	<0.001	0.195	0.091	0.376	0.001	-0.158	0.172	-0.123	0.289
Idaho	-0.357	0.003	-0.304	0.014	-0.300	0.015	-0.013	0.920	0.000	0.999	0.230	0.065
Illinois	-0.191	0.041	0.036	0.703	0.349	<0.001	0.115	0.222	-0.003	0.978	0.243	0.009
Indiana	-0.411	<0.001	-0.513	<0.001	0.213	0.073	0.444	<0.001	0.009	0.942	0.000	0.997
Iowa	-0.303	0.011	-0.357	0.002	-0.039	0.749	0.150	0.215	0.053	0.663	0.130	0.285
Kansas	-0.181	0.131	-0.271	0.022	0.075	0.533	0.156	0.194	0.119	0.325	-0.037	0.759
Kentucky	-0.339	0.003	-0.517	<0.001	0.211	0.075	0.404	<0.001	0.242	0.039	0.143	0.229
Louisiana	-0.303	0.012	-0.625	<0.001	0.329	0.007	0.410	0.001	0.110	0.371	0.118	0.339
Maine	-0.276	0.025	-0.345	0.005	0.280	0.024	-0.105	0.404	-0.027	0.831	0.107	0.392
Maryland	-0.371	0.001	-0.430	<0.001	0.253	0.032	0.413	<0.001	0.108	0.365	-0.031	0.797
Massachusetts	-0.031	0.749	-0.232	0.016	0.130	0.183	0.234	0.016	0.092	0.344	0.070	0.476
Michigan	-0.338	0.005	-0.485	<0.001	0.354	0.003	0.126	0.309	0.079	0.520	0.236	0.052
Minnesota	-0.508	<0.001	-0.566	<0.001	0.161	0.175	0.263	0.026	-0.042	0.727	-0.167	0.157
Mississippi	-0.193	0.120	-0.465	<0.001	0.599	<0.001	0.453	<0.001	0.229	0.064	0.188	0.130
Missouri	-0.189	0.114	-0.243	0.041	0.102	0.396	0.211	0.077	0.145	0.228	0.077	0.522
Montana	-0.18	0.150	-0.164	0.192	-0.218	0.081	-0.094	0.458	0.040	0.749	0.243	0.051
Nebraska	-0.281	0.016	-0.442	<0.001	0.078	0.510	0.342	0.003	0.028	0.816	-0.054	0.653
Nevada	-0.499	<0.001	-0.408	<0.001	0.457	<0.001	0.403	<0.001	-0.188	0.110	-0.217	0.064
New Hampshire	-0.229	0.045	-0.368	0.001	0.177	0.124	0.255	0.025	-0.104	0.369	-0.078	0.492
New Jersey	-0.207	0.076	-0.330	0.004	0.180	0.129	0.359	0.002	0.144	0.222	0.088	0.454
New Mexico	-0.454	<0.001	-0.635	<0.001	0.287	0.017	0.512	<0.001	-0.038	0.760	-0.235	0.054
New York	-0.243	0.033	-0.357	0.001	0.176	0.128	0.358	0.002	0.000	0.999	-0.089	0.440
North Carolina	-0.288	0.012	-0.434	<0.001	0.216	0.063	0.397	<0.001	-0.073	0.529	-0.122	0.249
North Dakota	-0.386	0.001	-0.405	0.001	0.289	0.019	0.148	0.237	0.128	0.307	0.047	0.706
Ohio	-0.201	0.097	-0.365	0.002	0.283	0.020	0.093	0.453	-0.015	0.906	0.036	0.770
Oklahoma	-0.254	0.131	-0.359	0.002[a]	0.146	0.220	0.312	0.008	-0.149	0.213	-0.029	0.811
Oregon	-0.495	<0.001	-0.458	<0.001	0.258	0.022	0.255	0.024	-0.141	0.215	-0.093	0.417
Pennsylvania	-0.388	0.001	-0.421	<0.001	0.263	0.026	0.388	0.001	0.148	0.212	-0.030	0.803
Rhode Island	-0.189	0.098	-0.404	<0.001	0.247	0.030	0.325	0.004	-0.079	0.490	-0.138	0.229
South Carolina	-0.392	0.001	-0.510	<0.001	0.393	0.001	0.442	<0.001	-0.155	0.192	-0.269	0.022
South Dakota	-0.217	0.076	-0.440	<0.001	-0.026	0.830	0.308	0.011	0.071	0.566	0.005	0.968
Tennessee	-0.492	<0.001	-0.506	<0.001	0.408	<0.001	0.457	<0.001	0.202	0.085	0.242	0.038
Texas	-0.538	<0.001	-0.555	<0.001	0.503	<0.001	0.476	<0.001	-0.054	0.647	0.149	0.206
Utah	-0.383	0.001	-0.485	<0.001	-0.030	0.803	0.311	0.008	0.074	0.537	-0.128	0.283
Vermont	-0.511	<0.001	-0.339	0.004	0.319	0.007	0.127	0.294	-0.037	0.758	-0.022	0.856
Virginia	-0.295	0.013	-0.328	0.005	0.346	0.003	0.217	0.072	0.013	0.915	-0.063	0.064
Washington	-0.437	<0.001	-0.315	0.001	-0.130	0.164	-0.080	0.393	0.179	0.152	0.143	0.124
West Virginia	0.04	0.760	-0.220	0.091	0.389	0.002	0.400	0.002	-0.075	0.570	-0.001	0.993
Wisconsin	-0.295	0.014	-0.390	0.001	0.126	0.308	0.123	0.316	-0.035	0.773	0.268	0.026
Wyoming	-0.14	0.262	-0.140	0.263	0.148	0.236	0.188	0.130	-0.024	0.845	0.235	0.057

Average temperature (1/2 Week) indicates the Pearson's correlation between daily average temperature

(℃) within 24 hours and the matched 7-day-delayed/14-day-delayed disseminating velocity of COVID-19. Average wind speed (1/2 Week) indicates the Pearson's correlation between daily average wind speed (m/s) within 24 hours and the matched 7-day-delayed/14-day-delayed disseminating velocity of COVID-19. Average air pressure (1/2 Week) indicates the Pearson's correlation between daily average air pressure (hPa) within 24 hours and the matched 7-day-delayed/14-day-delayed disseminating velocity of COVID-19.

Abbreviations: COVID-19, coronavirus disease 2019.

[a] P values indicate differences of Pearson correlations. $P < 0.1$ was considered statistically significant.

Supplementary Table 7. Secondary Pearson's Correlation between COVID-19 Clades and Coefficients of Climate Factors.

	L Clade		S Clade		O Clade		V Clade		G Clade		GR Clade		GH Clade		Total G Clade	
	Correlation	P value [a]	Correlation	P value [a]	Correlation	P value [a]	Correlation	P value [a]	Correlation	P value [a]	Correlation	P value [a]	Correlation	P value [a]	Correlation	P value [a]
**Worldwide**																
Average Temperature (1 Week)	-0.392	0.071	0.122	0.587	-0.341	0.120	-0.042	0.852	0.298	0.177	0.405	0.061	-0.173	0.440	0.381	0.080
Average Temperature (2 Week)	-0.431	0.045	0.142	0.527	-0.338	0.123	-0.006	0.978	0.237	0.288	0.406	0.061	-0.121	0.593	0.382	0.080
Average Wind Speed (1 Week)	-0.090	0.689	0.016	0.944	-0.397	0.067	0.512	0.015	0.232	0.299	0.187	0.406	-0.021	0.926	0.270	0.225
Average Wind Speed (2 Week)	-0.063	0.779	-0.129	0.566	0.010	0.966	0.597	0.003	0.055	0.809	-0.011	0.961	0.085	0.707	0.016	0.942
Average Air Pressure (1 Week)	0.513	0.061	0.266	0.357	0.103	0.726	-0.267	0.356	-0.665	0.009	-0.117	0.690	0.117	0.691	-0.435	0.120
Average Air Pressure (2 Week)	0.438	0.117	0.221	0.447	0.266	0.357	-0.463	0.095	-0.384	0.176	-0.221	0.447	0.041	0.889	-0.390	0.169
**The United States of America**																
Average Temperature (1 Week)	0.225	0.224	-0.099	0.598	0.147	0.431	-0.514	0.003	-0.371	0.040	-0.018	0.921	0.244	0.185	0.028	0.882
Average Temperature (2 Week)	0.154	0.409	0.111	0.554	0.056	0.766	-0.310	0.089	-0.210	0.257	-0.046	0.807	0.017	0.929	-0.125	0.502
Average Wind Speed (1 Week)	-0.082	0.662	-0.266	0.149	-0.071	0.706	-0.406	0.023	-0.137	0.462	-0.052	0.783	0.422	0.018	0.357	0.049
Average Wind Speed (2 Week)	0.158	0.396	-0.219	0.236	0.134	0.472	-0.062	0.740	0.205	0.268	-0.365	0.043	0.057	0.763	0.090	0.631
Average Air Pressure (1 Week)	-0.068	0.717	0.185	0.319	-0.099	0.595	0.053	0.776	-0.137	0.463	0.087	0.640	-0.041	0.828	-0.106	0.572
Average Air Pressure (2 Week)	-0.111	0.551	0.076	0.685	0.163	0.381	-0.366	0.043	-0.160	0.391	0.112	0.550	0.035	0.850	-0.031	0.869

Average temperature (1/2 Week) indicates the Pearson's correlation between daily average temperature (℃) within 24 hours and the matched 7-day-delayed/14-day-delayed disseminating velocity of COVID-19. Average wind speed (1/2 Week) indicates the Pearson's correlation between daily average wind speed (m/s) within 24 hours and the matched 7-day-delayed/14-day-delayed disseminating velocity of COVID-19. Average air pressure (1/2 Week) indicates the Pearson's correlation between daily average air pressure (hPa) within 24 hours and the matched 7-day-delayed/14-day-delayed disseminating velocity of COVID-19.

Abbreviations: COVID-19, coronavirus disease 2019.

[a] P values indicate differences of Pearson correlations. $P < 0.1$ was considered statistically significant.

Supplementary Table 8. Secondary Pearson's Correlation between COVID-19 Clades and Altitude/Latitude Worldwide and in USA.

	L Clade		S Clade		O Clade		V Clade		G Clade		GR Clade		GH Clade		Total G Clade	
	Correlation	P value[a]	Correlation	P value[a]	Correlation	P value[a]	Correlation	P value[a]	Correlation	P value[a]	Correlation	P value[a]	Correlation	P value[a]	Correlation	P value[a]
**Worldwide**																
Latitude	-0.051	0.823	-0.293	0.186	-0.222	0.322	0.363	0.097	0.290	0.191	0.049	0.827	0.129	0.566	0.295	0.183
Altitude	-0.079	0.727	0.175	0.436	0.495	0.019	-0.180	0.423	-0.273	0.219	-0.442	0.040	0.165	0.462	-0.401	0.064
**The United States of America**																
Latitude	-0.018	0.925	-0.262	0.154	0.000	1.000	0.650	<0.001	0.390	0.030	-0.088	0.637	-0.082	0.659	0.130	0.486
Altitude	0.039	0.836	-0.174	0.348	0.599	<0.001	-0.256	0.165	0.042	0.823	-0.200	0.282	-0.029	0.877	-0.060	0.750

Abbreviations: COVID-19, coronavirus disease 2019.

[a] P values indicate differences of Pearson correlations. $P < 0.1$ was considered statistically significant.

Supplementary Table 9. Cluster Analysis of COVID-19 Clades, Pandemic, Average Temperature, and Average Wind Speed in USA.

States	G Clade[a]	GR Clade[a]	GH Clade[a]	L Clade[a]	S Clade[a]	O Clade[a]	V Clade[a]	Total G Clade	Genome Cluster	Pandemic Cluster	Average Temperature Cluster	Average Wind Speed Cluster	Average Air Pressure Cluster
Alabama	/	/	/	/	/	/	/	/	/	I	C	A	A
Alaska	33.33%	3.33%	50.00%	0.00%	0.00%	0.00%	13.33%	86.67%	C	A	B	C	B
Arizona	3.53%	8.24%	72.94%	0.00%	12.94%	0.00%	2.35%	84.71%	A	F	B	C	B
Arkansas	/	/	/	/	/	/	/	/	/	E	A	A	A
California	7.77%	5.25%	38.95%	23.92%	15.34%	8.07%	0.71%	51.97%	D	C	D	A	A
Colorado	12.50%	0.00%	37.50%	12.50%	0.00%	37.50%	0.00%	50.00%	E	A	A	A	A
Connecticut	7.69%	6.79%	71.49%	0.00%	12.22%	0.45%	1.36%	85.97%	A	A	C	C	D
DC	/	/	/	/	/	/	/	/	/	G	C	A	A
Florida	5.06%	3.16%	72.78%	0.00%	17.09%	1.27%	0.63%	81.01%	A	C	C	A	A
Georgia	0.00%	16.13%	22.58%	0.00%	54.84%	6.45%	0.00%	38.71%	F	C	C	B	B
Idaho	10.34%	0.00%	87.93%	0.00%	0.00%	0.00%	1.72%	98.28%	A	A	B	B	B
Illinois	17.69%	0.77%	40.77%	3.85%	33.08%	3.85%	0.00%	59.23%	B	C	C	A	A
Indiana	42.86%	0.00%	42.86%	0.00%	7.14%	7.14%	0.00%	85.71%	C	C	C	B	B
Iowa	39.29%	0.00%	60.71%	0.00%	0.00%	0.00%	0.00%	100.00%	C	C	A	B	B
Kansas	/	/	/	/	/	/	/	/	/	A	A	A	A
Kentucky	/	/	/	/	/	/	/	/	/	C	C	A	A
Louisiana	4.79%	0.00%	94.93%	0.00%	0.28%	0.00%	0.00%	99.72%	A	A	C	A	A
Maine	/	/	/	/	/	/	/	/	/	A	C	D	D
Maryland	2.97%	13.86%	61.39%	0.00%	18.81%	0.99%	1.98%	78.22%	A	C	C	A	A
Massachusetts	6.07%	3.16%	82.28%	0.00%	7.28%	0.49%	0.73%	91.50%	A	C	C	B	B
Michigan	10.18%	1.52%	82.83%	0.30%	4.41%	0.30%	0.46%	94.53%	A	A	C	A	A
Minnesota	6.32%	6.32%	61.26%	1.98%	15.02%	4.35%	4.74%	73.91%	A	A	A	A	A
Mississippi	/	/	/	/	/	/	/	/	/	A	C	A	A
Missouri	/	/	/	/	/	/	/	/	/	C	A	C	D
Montana	/	/	/	/	/	/	/	/	/	A	A	A	A
Nebraska	2.50%	20.00%	70.00%	5.00%	2.50%	0.00%	0.00%	92.50%	A	A	A	D	E
Nevada	/	/	/	/	/	/	/	/	/	C	B	B	B
New Hampshire	/	/	/	/	/	/	/	/	/	C	C	A	A
New Jersey	0.00%	2.38%	90.48	2.38%	2.38%	0.00%	2.38%	92.86	A	C	C	B	B

			%					%					
New Mexico	4.26%	5.67%	82.27%	0.00%	6.38%	1.42%	0.00%	92.20%	A	A	B	D	C
New York	3.98%	3.92%	83.12%	0.14%	2.90%	2.23%	3.71%	91.02%	A	C	C	A	A
North Carolina	1.61%	0.00%	74.19%	0.00%	24.19%	0.00%	0.00%	75.81%	A	C	C	A	A
North Dakota	/	/	/	/	/	/	/	/	/	B	A	B	B
Ohio	7.14%	7.14%	75.00%	0.00%	7.14%	0.00%	3.57%	89.29%	A	A	C	A	A
Oklahoma	/	/	/	/	/	/	/	/	/	C	A	A	A
Oregon	21.92%	4.11%	54.79%	0.00%	6.85%	2.74%	9.59%	80.82%	C	C	B	A	A
Pennsylvania	5.41%	6.31%	81.08%	0.00%	6.31%	0.90%	0.00%	92.79%	A	C	C	A	A
Rhode Island	/	/	/	/	/	/	/	/	/	D	C	C	D
South Carolina	10.00%	0.00%	30.00%	0.00%	60.00%	0.00%	0.00%	40.00%	F	C	C	A	A
South Dakota	/	/	/	/	/	/	/	/	/	D	A	D	B
Tennessee	/	/	/	/	/	/	/	/	/	C	C	A	A
Texas	15.04%	5.31%	49.85%	0.29%	27.43%	0.59%	1.47%	70.21%	B	C	C	A	A
Utah	29.75%	1.58%	58.54%	0.95%	7.91%	0.95%	0.32%	89.87%	C	A	B	B	B
Vermont	/	/	/	/	/	/	/	/	/	C	C	A	A
Virginia	6.01%	9.01%	76.88%	0.00%	6.01%	0.00%	2.10%	91.89%	A	A	C	A	A
Washington	2.92%	4.17%	41.36%	0.00%	47.96%	2.87%	0.72%	48.45%	F	A	B	A	A
West Virginia	/	/	/	/	/	/	/	/	/	H	C	C	B
Wisconsin	17.16%	2.41%	37.27%	27.61%	12.06%	2.95%	0.54%	56.84%	D	A	C	D	C
Wyoming	5.43%	0.00%	66.30%	0.00%	11.96%	16.30%	0.00%	71.74%	A	A	A	D	C

Pandemic cluster indicates cluster analysis according to daily disseminating velocity. Average temperature indicates daily average temperature (℃) within 24 hours. Average wind speed indicates daily average wind speed (m/s) within 24 hours. Average air pressure indicates daily average air pressure (hPa) within 24 hours.

Abbreviations: COVID-19, coronavirus disease 2019; DC, Washington District of Columbia.

[a]The proportions of different clades of COVID-19 were collected from GISAID (Global Initiative on Sharing All Influenza Data) as of June 10th, 2020.[2]

Supplementary Table 10. Intra-cluster Analysis in Epidemic Disseminating Velocity Cluster and COVID-19 Genome Cluster in USA.

**Epidemic Disseminating Velocity Cluster**			
	Cluster A Median Coefficients (IQR)	Cluster C Median Coefficients (IQR)	P value[a]
Average Temperature (1 Week)	-0.295 (-0.383 – -0.193)	-0.329 (-0.431 – -0.224)	0.464
Average Temperature (2 Week)	-0.390 (-0.485 – -0.304)	-0.407 (-0.477 – -0.337)	0.902
Average Wind Speed (1 Week)	0.013 (-0.027 – 0.110)	-0.020 (-0.113 – 0.117)	0.240
Average Wind Speed (2 Week)	0.087 (-0.063 – 0.230)	-0.030 (-0.100 – 0.099)	0.219
Average Air Pressure (1 Week)	0.161 (0.075 – 0.287)	0.223 (0.177 – 0.333)	0.122
Average Air Pressure (2 Week)	0.156 (0.342 – 0.512)	0.366 (0.228 – 0.406)	0.014
**COVID-19 Genome Cluster**			
	Cluster A Median Coefficients (IQR)	Cluster C Median Coefficients (IQR)	P value[a]
Average Temperature (1 Week)	-0.292 (-0.361 – -0.206)	-0.411 (-0.603 – -0.343)	0.016
Average Temperature (2 Week)	-0.385 (-0.453 – -0.286)	-0.485 (-0.621 – -0.408)	0.076
Average Wind Speed (1 Week)	0.007 (-0.039 – 0.109)	0.009 (-0.161 – 0.064)	0.356
Average Wind Speed (2 Week)	-0.034 (-0.102 – 0.096)	0.000 (-0.111 – 0.109)	0.913
Average Air Pressure (1 Week)	0.211 (0.158 – 0.297)	0.211 (-0.035 – 0.236)	0.304
Average Air Pressure (2 Week)	0.303 (0.145 – 0.390)	0.255 (0.034 – 0.378)	0.602

Average temperature (1/2 Week) indicates the Pearson's correlation between daily average temperature (℃) within 24 hours and the matched 7-day-delayed/14-day-delayed disseminating velocity of COVID-19. Average wind speed (1/2 Week) indicates the Pearson's correlation between daily average wind speed (m/s) within 24 hours and the matched 7-day-delayed/14-day-delayed disseminating velocity of COVID-19. Average air pressure (1/2 Week) indicates the Pearson's correlation between daily average air pressure (hPa) within 24 hours and the matched 7-day-delayed/14-day-delayed disseminating velocity of COVID-19.

Abbreviations: COVID-19, coronavirus disease 2019.

[a] P values indicate differences between different clusters and were calculated by Mann-Whitney U test. $P < 0.05$ was considered statistically significant.

Supplementary Figure 1. Pearson's correlation between the meteorological factors and the matched disseminating velocity of COVID-19 worldwide (1-week dataset)

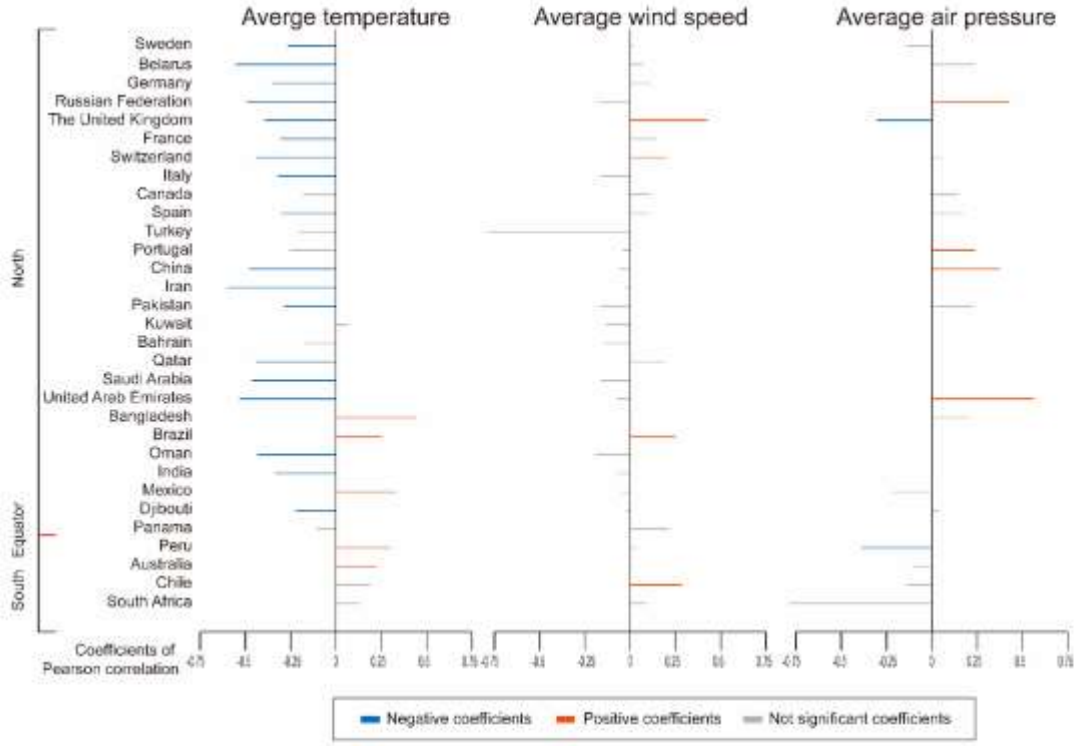

The orange and blue lines depict the positive and negative coefficients of Pearson correlation between the meteorological factors and the matched disseminating velocity of COVID-19 with p-value < 0.05. The grey lines depict the coefficients of Pearson correlation with no statistical significance (p-value ≥ 0.05).

Supplementary Figure 2. Atlas for distributions of virus clades for USA and Atlas for coefficients of correlation in Pearson analysis for USA (1-week delayed matching of data).

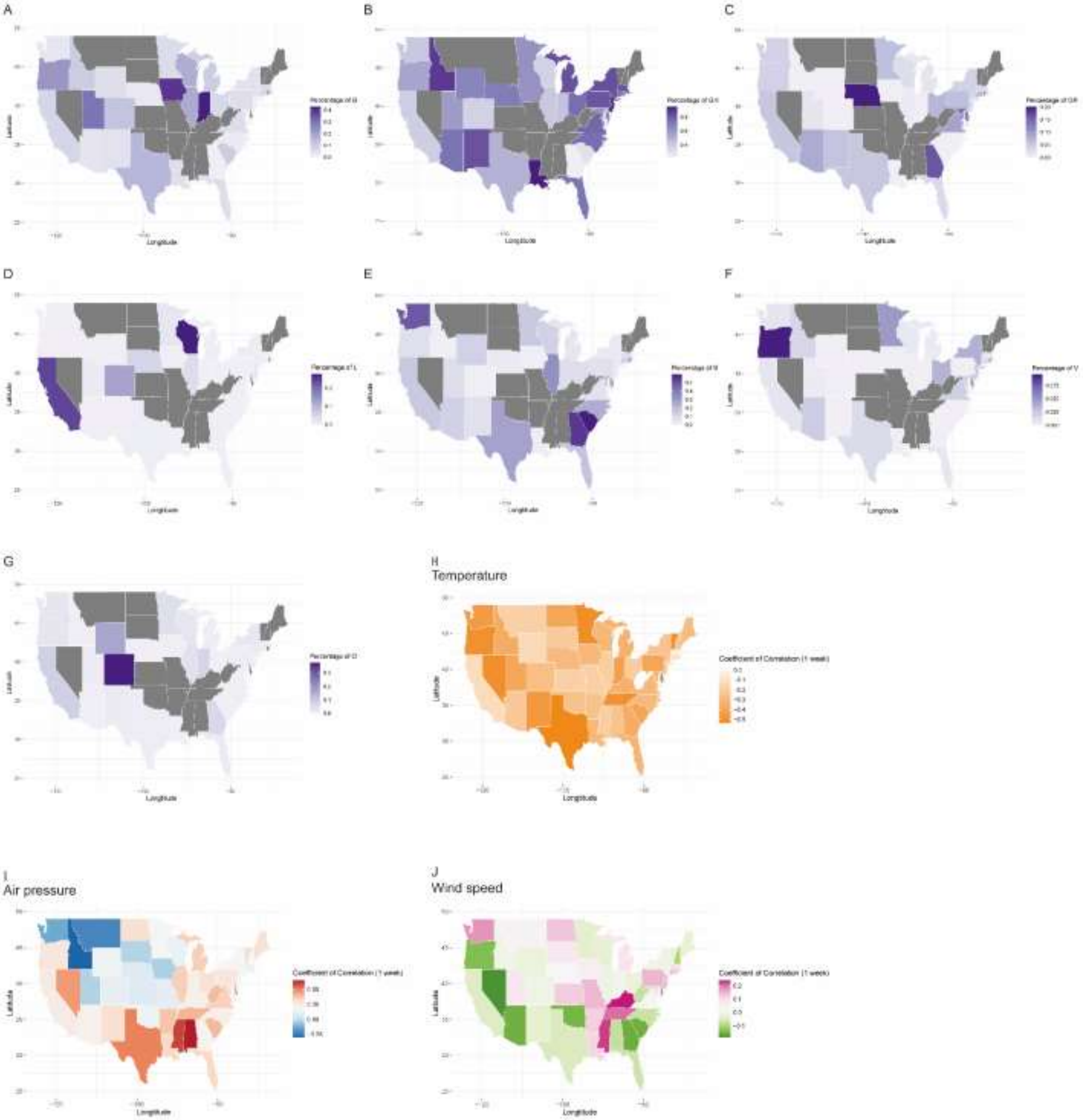

Proportions of clades of states in USA are shown in the map (A-G). Values of proportion are referring to color scale bar. Grey color is representing to absence of genome data in the corresponding states.

The coefficients of correlation of daily meteorological estimates versus daily pandemic data are shown in H-J. H for coefficient of average air temperature in 24 hours versus daily calculated disseminating velocity of virus, I and J for that of average wind speed and average air pressure respectively. Values of coefficient are referring to color scale bar.

Supplementary Figure 3. Line chart for temperature cluster A and C.

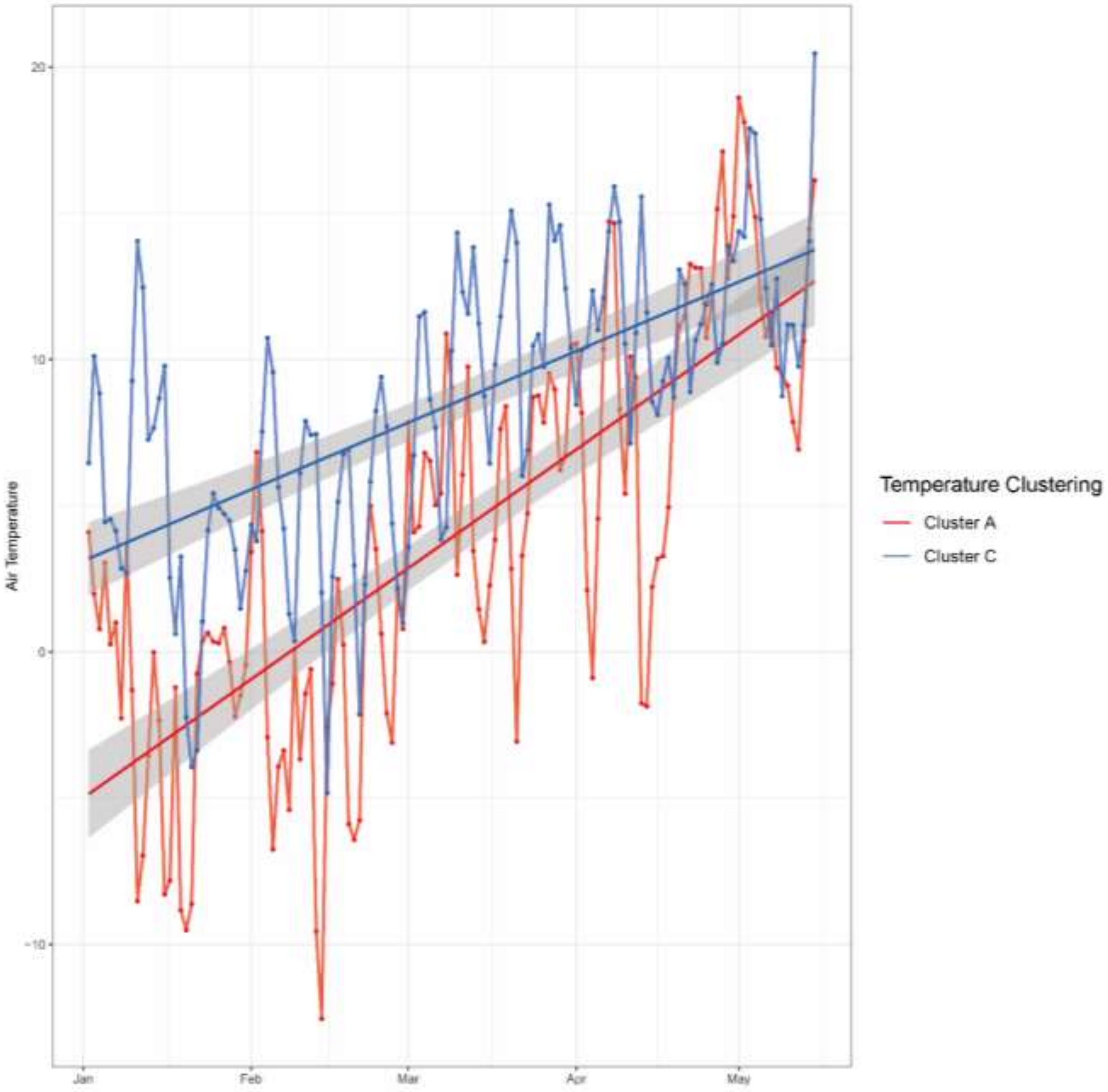

The line chart shows average air temperature records of all states in temperature cluster A and C, respectively.

Reference

1. World Health Organization. WHO Coronavirus Disease (COVID-19) Dashboard. https://covid19.who.int/. Accessed June 10th,2020.
2. GISAID. Global Initiative on Sharing All Influenza Data. https://www.gisaid.org/. Accessed June 10th,2020.
